# Contrast-enhanced, conebeam CT-based, fractionated radiotherapy and follow-up monitoring of orthotopic mouse glioblastoma: a proof-of-concept study

**DOI:** 10.1186/s13014-020-1470-2

**Published:** 2020-01-22

**Authors:** Benjamin Stegen, Alexander Nieto, Valerie Albrecht, Jessica Maas, Michael Orth, Klement Neumaier, Sabine Reinhardt, Moritz Weick-Kleemann, Wilfried Goetz, Merle Reinhart, Katia Parodi, Claus Belka, Maximilian Niyazi, Kirsten Lauber

**Affiliations:** 1 Department of Radiation Oncology, University Hospital, Ludwig-Maximilians-Universität München, Marchioninistrasse 15, 81377 Munich, Germany; 2 German Cancer Consortium (DKTK) partnersite Munich, Munich, Germany; 3 German Cancer Research Center (DKFZ), Heidelberg, Germany; 4 Department of Medical Physics, Ludwig-Maximilians-Universität München, Munich, Germany; 5 X-Strahl Inc., Suwanee, GA USA; 6 Clinical Cooperation Group ‘Personalized Radiotherapy in Head and Neck Cancer’ Helmholtz Center Munich, German Research Center for Environmental Health GmbH, Neuherberg, Germany

**Keywords:** Small animal tumor models, Preclinical radiotherapy, Molecular radiation oncology, Orthotopic glioblastoma, Samll animal radiotherapy, Small animal radiation platforms

## Abstract

**Background:**

Despite aggressive treatment regimens comprising surgery and radiochemotherapy, glioblastoma (GBM) remains a cancer entity with very poor prognosis. The development of novel, combined modality approaches necessitates adequate preclinical model systems and therapy regimens that closely reflect the clinical situation. So far, image-guided, fractionated radiotherapy of orthotopic GBM models represents a major limitation in this regard.

**Methods:**

GL261 mouse GBM cells were inoculated into the right hemispheres of C57BL/6 mice. Tumor growth was monitored by contrast-enhanced conebeam CT (CBCT) scans. When reaching an average volume of approximately 7 mm^3^, GBM tumors were irradiated with daily fractions of 2 Gy up to a cumulative dose of 20 Gy in different beam collimation settings. For treatment planning and tumor volume follow-up, contrast-enhanced CBCT scans were performed twice per week. Daily repositioning of animals was achieved by alignment of bony structures in native CBCT scans. When showing neurological symptoms, mice were sacrificed by cardiac perfusion. Brains, livers, and kidneys were processed into histologic sections. Potential toxic effects of contrast agent administration were assessed by measurement of liver enzyme and creatinine serum levels and by histologic examination.

**Results:**

Tumors were successfully visualized by contrast-enhanced CBCT scans with a detection limit of approximately 2 mm^3^, and treatment planning could be performed. For daily repositioning of the animals, alignment of bony structures in native CT scans was well feasible. Fractionated irradiation caused a significant delay in tumor growth translating into significantly prolonged survival in clear dependence of the beam collimation setting and margin size. Brain sections revealed tumors of similar appearance and volume on the day of euthanasia. Importantly, the repeated contrast agent injections were well tolerated, as liver enzyme and creatinine serum levels were only subclinically elevated, and liver and kidney sections displayed normal histomorphology.

**Conclusions:**

Contrast-enhanced, CT-based, fractionated radiation of orthotopic mouse GBM represents a versatile preclinical technique for the development and evaluation of multimodal radiotherapeutic approaches in combination with novel therapeutic agents in order to accelerate translation into clinical testing.

## Introduction

Since the approval of concomitant and adjuvant temozolomide as addition to radiotherapy for the treatment of glioblastoma (GBM) [1], intensive efforts have been spent in order to identify novel therapeutic strategies to improve the outcome of this devastating disease. The obvious treatment resistance of GBM is mainly attributed to its particular heterogeneity with a strong impact of cancer stem cells and its unique and protective tumor microenvironment, including excessively distorted tumor vasculature and resulting hypoxia [2]. Patient cohort analyses and unbiased screening approaches have identified various candidates for specific therapeutic targeting. However, prior to clinical evaluation, these approaches require preclinical testing – ideally in addition and/or in comparison to the present clinical standard. Fractionated radiotherapy of orthotopic glioblastoma models with feasible and robust follow-up monitoring still represents a major challenge in this regard, although dedicated small animal radiotherapy platforms are commercially available and have been utilized for the treatment of orthotopic glioblastoma by various groups [3–7]. Nevertheless, systematic analyses with classically fractionated radiation protocols are scarce [8, 9], and reliable tumor localization as well as tumor volume follow-up are often hampered by limited access to magnetic resonance (MR), positron emission tomography (PET), or bioluminescence (BL) imaging platforms.

In this study, we describe a fractionated radiotherapy protocol (2 weeks with 5 × 2 Gy) for orthotopic mouse glioblastoma with implementation of contrast-enhanced CBCT scans for tumor localization and volume follow-up. Our work was performed using a commercial small animal radiotherapy platform only and can be adapted to virtually any kind of combined modality treatment approach with biologically targeted and/or immunotherapeutic agents.

## Materials and methods

### Depth dose measurements with thermoluminescence dosimeters and radiochromic films

**Fig. 1:**
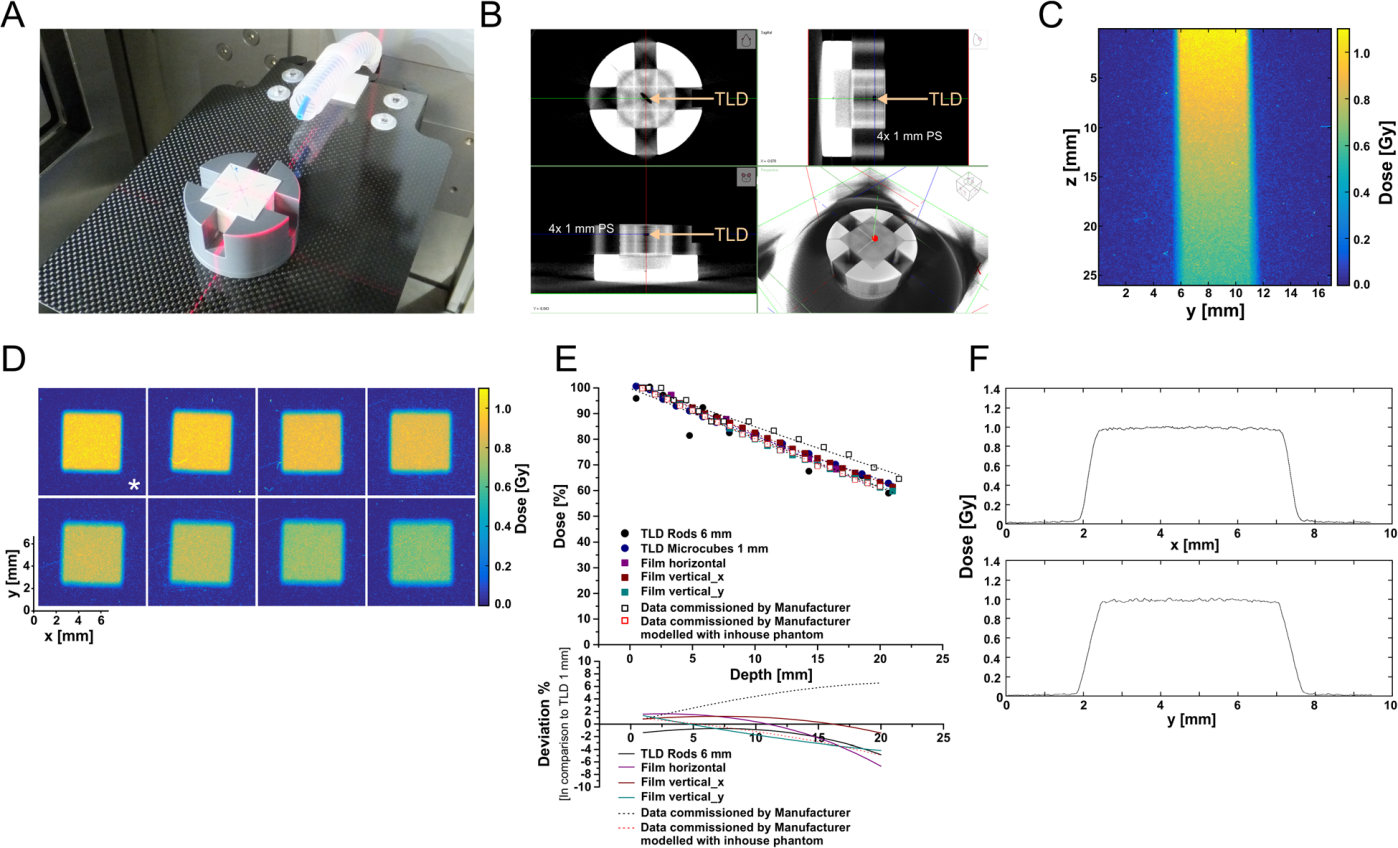
Depth dose measurements on the small animal radiotherapy platform. **a** The used mouse surrogate phantom consisting of a series of 1 mm polystyrene (PS) slices with fitted cavities to carry either rod-like or microcube-type thermoluminescence dosimeters (TLDs), or GAFchromic EBT3 dosimetry films, respectively. **b** A conebeam CT (CBCT) scan of the phantom shown in (**a**). **c** Acquisition of depth dose data in the continuous, vertical film positioning mode. **d** Acquisition of depth dose data in the horizontal film positioning mode. The asterisk indicates the film from which the penumbra data in (**f**) were extracted. **e** Depth dose curves obtained with the phantom shown in (**a**, **b**), for 5× 5 mm^2^ beam collimation, and the indicated dosimetric devices in comparison to commissioning data provided by the manufacturer (as measured by the manufacturer and calculated with the point dose calculator tool PDC 1.2 (X-Strahl) modelling the inhouse phantom used). The lower graph depicts the deviation in dose [%] as determined by the different detection methods in comparison to 1 mm^3^ microcube TLDs. **f** The lateral penumbra of the irradiation beam with 5× 5 mm^2^ collimation in x- and y-direction as extracted from the film marked with an asterisk in (**d**)

Depth dose measurements were performed in a polystyrene phantom (26× 26× 22 mm^3^, L× W× H, Fig. 1a and b) using thermoluminescence dosimeters (TLDs) with rod-like (Ø = 1 mm, l = 6 mm) or microcube-like (1× 1× 1 mm^3^) shape (TLD-100, LiF:Mg, Ti, Thermo Scientific, Schwerte, Germany) and radiochromic films (GAFchromic EBT3, Ashland, Bridgewater, NJ, USA). The phantom consisted of a set of polystyrene plates with 1 mm thickness. For each type of TLDs (rods and microcubes), a polystyrene carrier plate with precisely fitting center cavity was designed. This allowed exact positioning of the TLD in steps of 1 mm depth from 0.5–20.5 mm.

For the film measurements, the phantom was used without the TLD carrier plate. The discrete depth dose measurements were performed in horizontal orientation of the film in steps of 1 mm (0–20 mm depth). For the continuous measurements, the films were positioned in the center of the plate stack. All measurements were executed with constant source-to-surface distance (SSD = 350 mm). TLD analyses were performed on a Thermoluminescence Detector 2000A (Mirion Technologies, Munich, Germany) in combination with a Harshaw TLD-Analyzer 2080 (Thermo Scientific) [10]. EBT3 films were digitized 48 h after irradiation with a flat-bed scanner (Epson Perfection V700 Photo, 1200 dpi, 48-bit RGB, Epson, Meerbusch, Germany). Subsequently, images were background corrected, and pixel values were converted into dose values. Calibration settings were acquired at a 6 MV-photon irradiation machine with the single channel method. Discrete dose values were then computed as means of at least 120 single values in homogeneous dose areas in the center of the dose images.

### Animals

All animal experiments were performed according to the FELASA guidelines and upon ethical approval by the *Regierung von Oberbayern* including a priori group size estimations (effect size 2.0, alpha error 0.05, beta error 0.2) in order to prevent underpowering. Female C57BL/6 mice (8–10 weeks old, in total *n* = 29) were obtained from Charles River (Sulzfeld, Germany) and were housed in groups of 4 animals in individually ventilated cages (GM500, Tecniplast, Hohenpeißenberg, Germany) in a specified pathogen-free animal facility with a 12 h day/night cycle. Standard rodent feed (Ssniff, Soest, Germany) and water were provided ad libitum. Animals were inspected daily and were sacrificed when reaching a pre-defined health score comprising critical weight loss, neurological symptoms, and overall health performance.

### Cells

GL261 murine glioblastoma cells (C57BL/6 background) were obtained from the National Cancer Institute (NCI, Frederick, MD, USA) and were cultured in DMEM supplemented with 10% heat-inactivated fetal calf serum (FCS), 100 units/ml penicillin, and 0.1 mg/ml streptomycin (all from Thermo Scientific) at 37 °C and 5% CO_2_ [11–13]. Negative testing for mycoplasma contamination was confirmed regularly. Cells were grown to subconfluent levels, detached by trypsinization (Thermo Scientific), and collected by centrifugation. Upon washing in phosphate-buffered saline (PBS, Thermo Scientific) cells were resuspended to a final concentration of 90,000 cells/μl. One microliter (90,000 cells) was used for inoculation.

### Intracranial implantation of tumor cells

Two hours prior to implantation mice were pre-medicated with 200 μg/g body weight metamizol (WDT, Garbsen, Germany) to control for postoperative pain and inflammation. Mice were anaesthetized with an intraperitoneal injection of 100 μg/g ketamine and 10 μg/g xylazine (both from WDT). Upon reaching surgical tolerance, the mouse head was mounted onto a stereotaxic frame (David Instruments, Tujanga, CA, USA) positioned on a heating plate with 37 °C. The skull was exposed by a 0.5 cm longitudinal skin incision, and a hole was drilled 1.5 mm lateral (right) and 1 mm posterior to the *bregma* using a pair of 23G and a 21G microlances (BD Biosciences, Heidelberg, Germany). With a stereotactically guided glass syringe (Hamilton, Bonaduz, Switzerland) 90,000 GL261 cells were injected in 1 μl in 3 mm depth from the *dura* surface into the right *striatum*. Injection was executed over a time period of 2 min, and the syringe was slowly withdrawn in 3–4 steps. The skin was closed using Ethibond Excel 5–0 suture material (Ethicon, Norderstedt, Germany), and mice were monitored on a heated pad until regaining consciousness.

### Contrast-enhanced computed tomography scans and fractionated irradiation

**Fig. 2:**
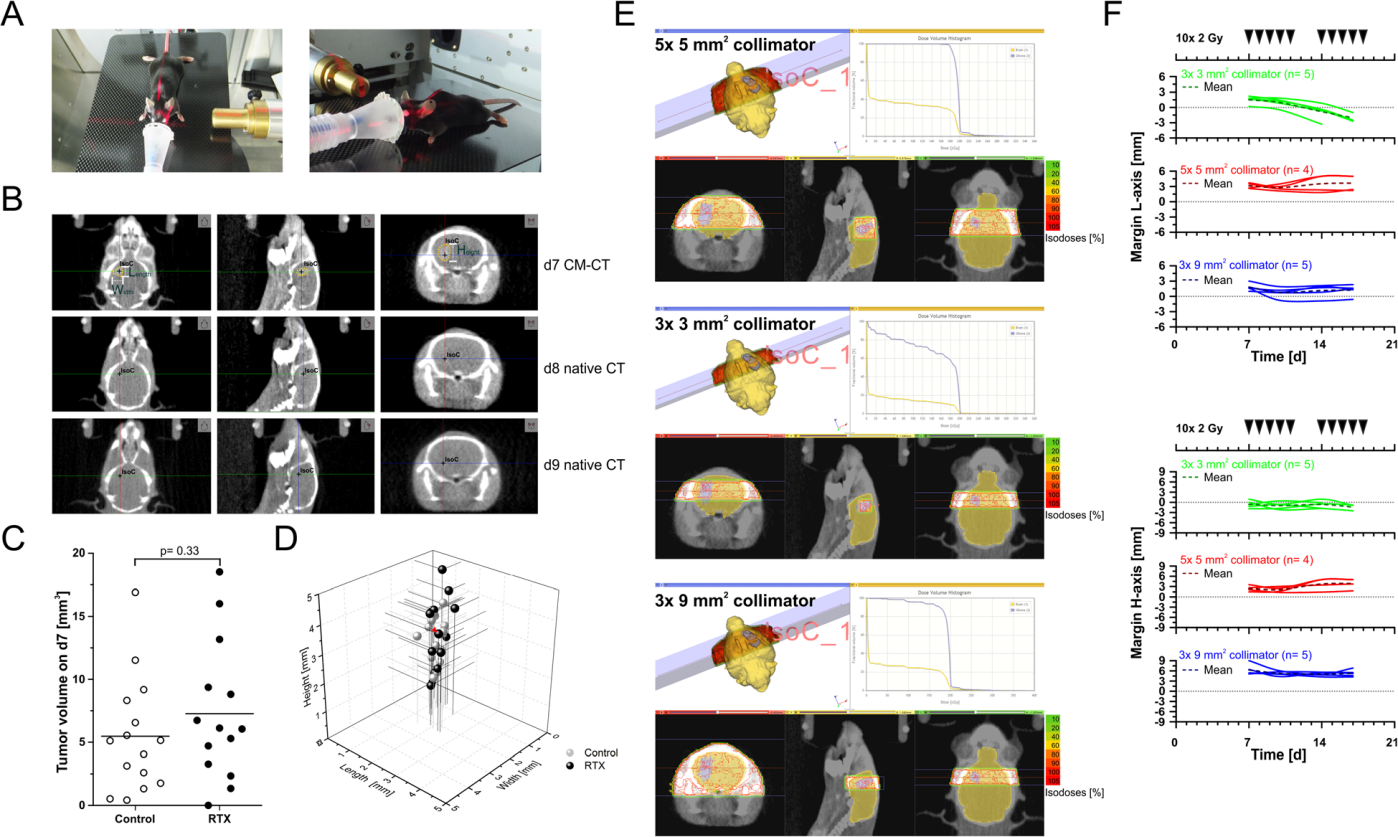
Contrast-enhanced and native CBCT scans for tumor localization, tumor volume follow-up, treatment planning, dose administration, and repositioning of animals. **a** Positioning and fixation of the mouse inside an anaesthetic mask with an elastic membrane. **b** Alternating contrast-enhanced (d7) and native CBCT scans (d8 and d9) for tumor localization, tumor volume follow-up, treatment planning, dose administration, and repositioning of animals. The black cross marks the isocenter defined as the center of the contrast-enriching volume on d7 and inferred from its relative position to bony structures in native CT scans on d8/d9. **c** Tumor volumes of irradiated and non-irradiated animals at the start of treatment (d7) as determined by Lx Hx W calculations shown in (**b**). *p*-value as calculated by exact Wilcoxon Rank test. **d** Tumor measures (L, H, and W) of individual animals at the start of treatment (d7). The red arrowhead indicates the animal shown in (**b**) and (**e**). **e** Treatment plans and dose-volume histograms for irradiation with two transversal, contralateral beams of 5 × 5, 3 × 3, or 3× 9 mm^2^ collimation, respectively. **f** Analyses of margins between contrast enriching tumor volumes and beam collimation settings for all irradiated animals in L- and H-axis over time

Starting at d7 after implantation, tumor growth was monitored by contrast-enhanced conebeam computed tomography (CBCT) scans twice weekly using a small animal radiation research platform (SARRP, X-Strahl, Camberley, Great Britain) [14]. Mice were anaesthetized with 2–3% isoflurane in oxygen and positioned in the SARRP unit on a fiberglass couch (Fig. 2a). The mouse head was positioned into an anaesthesia nosecone, and a CBCT scan was acquired with 360 projection images (1° per image) with X-ray tube settings of 60 kV and 0.8 mA, and 1.0 mm aluminium filter. In order to enhance soft tissue contrast, 300 μl imeron-300 (equivalent to 90 mg iodine, Bracco, Konstanz, Germany) were injected intravenously into the tail vein 3 min prior to CBCT acquisition on d7, d10, d14 and d17. On d8, d9, and d11, d15, d16, and d18 native CBCT scans were acquired for animal positioning prior to radiotherapy.

Radiotherapy was performed on d7-d11 and d14–18 in daily fractions of 2 Gy (in total 10 × 2 Gy) with two contralateral beams (gantry positions − 90° and 90°) of different collimation (fixed nozzle collimators of 3× 3 mm^2^, 5× 5 mm^2^ and 3× 9 mm^2^, X-ray tube settings 220 kV, 13 mA, 0.15 mm copper filter, Fig. 2e). Guided by the contrast-enhanced CBCT scans on d7, d10, d14, and d17 the isocenter of irradiation was aligned to the center of the contrast enriching tumor volume, and treatment planning was performed with Muriplan software (X-Strahl). On all other days, isocenter alignment was inferred in native CBCT scans from the relative position to bony structures (Fig. 2b).

### Determination of tumor volumes from CBCT scans

**Fig. 3:**
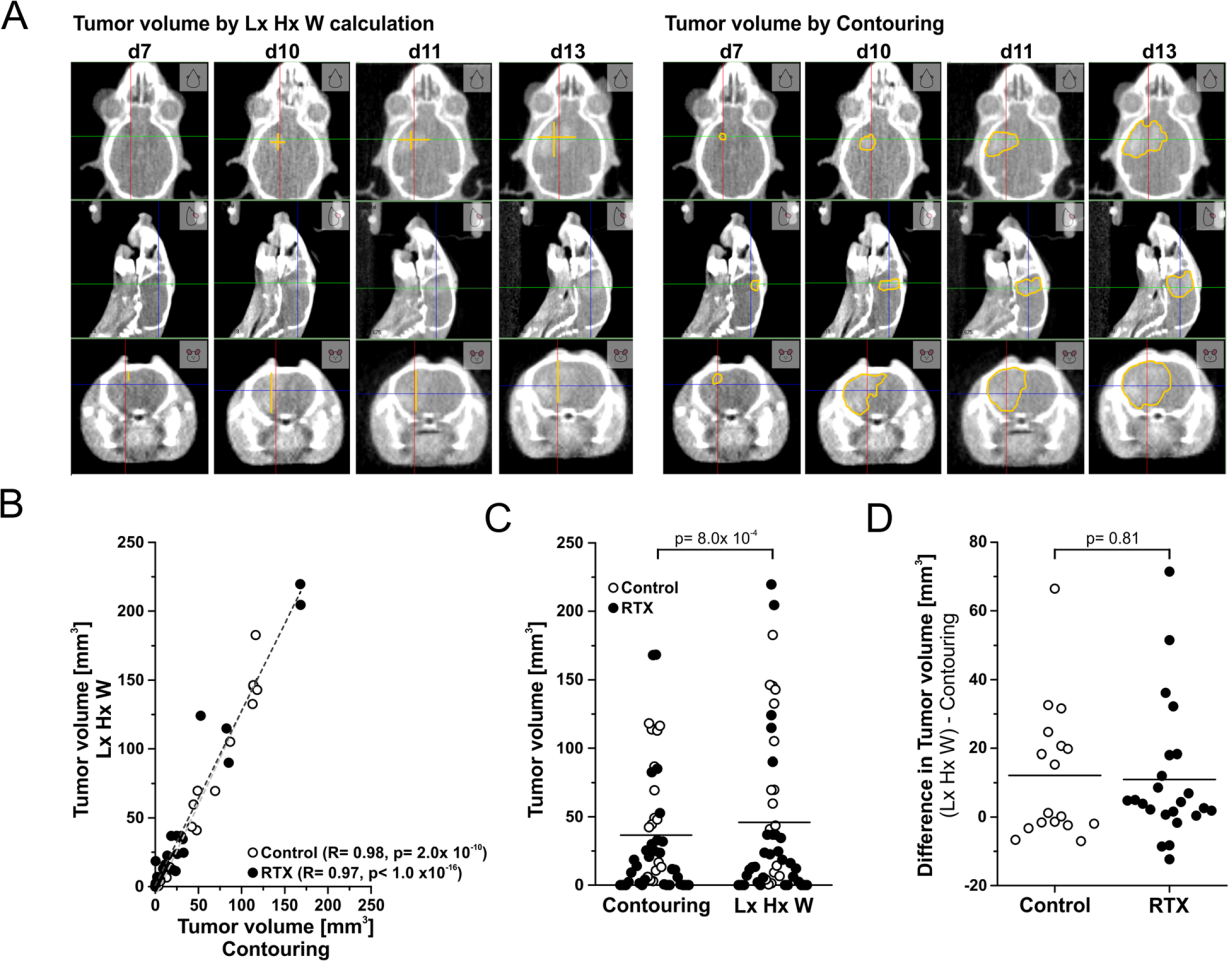
Measurement of tumor volumes by contrast-enhanced CBCT scans. **a** Comparison of tumor volume calculation methods using Lx Hx W calculation in MuriSlice software or manual contouring in ImageJ software, respectively. **b** Pearson correlation analyses of tumor volumes as assessed by Lx Hx W method or contouring for irradiated and non-irradiated animals. **c** Comparison of tumor volumes as determined by contouring or Lx Hx W method. *p*-value as calculated by paired Wilcoxon-Rank test. **d** Differences in tumor volumes as determined by Lx Hx W method or contouring. *p*-value as calculated by exact Wilcoxon Rank test

Tumor volumes were determined from CBCT scans by two approaches: Lx Hx W measurement of the 3 longest orthogonal axes (Fig. 2b) and via manual contouring with the segmentation editor tool of ImageJ software [15]. Results were subjected to correlation analyses according to the Pearson algorithm (Fig. 3b) and exact Wilcoxon Rank tests.

### Cardiovascular perfusion and fixation of organs

When reaching a pre-defined humane endpoint, mice were sacrificed by cardiovascular perfusion. Twenty minutes prior to median sternotomy, mice received 0.1 μg/g buprenorphin (Bayer, Leverkusen, Germany) subcutaneously. Anaesthesia (20 μg/g xylazin and 300 μg/g ketamin) was administered intraperitoneally, and upon reaching anaesthesia state IV, abdomen and thorax were opened. For cardiovascular perfusion, a peristaltic pump was used (operated at 8 ml/min), including a 3-way valve to switch between the infusion solutions. A small incision was made into the posterior end of the left ventricle, and a 20G (BD Biosciences) needle was inserted. After cutting a small outlet into the right auricle, mice were initially perfused with PBS controlled by lightening of the liver, followed by perfusion with 3.5% paraformaldehyde (Sigma-Aldrich, Taufkirchen, Germany) for further 2 min.

### Histology

After perfusion and explantation, brains, livers, and kidneys were kept in 3.5% paraformaldehyde for 48 h at 4 °C. Subsequently, brains were dehydrated in 30% sucrose for 48 h, embedded in Neg-50 frozen section medium (Thermo Scientific) and stored at − 80 °C until 40 μm tissue sections were prepared. Livers and kidneys were dehydrated in an increasing alcohol series (70, 80, 90, and 100%) and xylene, embedded in paraffin (all from Sigma-Aldrich), and processed into histological sections of 10 μm. All sections were stained with hematoxylin and eosin (H&E, Merck KGaA, Darmstadt, Germany) and evaluated by light microscopy with a ZEISS Axio Lab A1 microscope equipped with a ZEISS AxioCam ERc5s camera (Carl Zeiss, Oberkochen, Germany).

### Determination of ALT and creatinine serum levels

Potential toxic effects of contrast agent administration on livers and kidneys were assessed by measurement of alanine aminotransferase (ALT) and creatinine serum levels using colorimetric assay kits (Biovision, Ilmenau, Germany). Retrobulbar blood was drawn on d18 after tumor inoculation at the end of therapy. Untreated, naïve animals served as controls.

### Statistics

Statistical analyses were performed in Origin 9.1 Pro software. For group comparisons, unpaired or paired Wilcoxon Rank tests were applied, respectively, and correlation analyses were performed using the Pearson algorithm. Time-to-event analyses of CBCT progression and overall survival were conducted by Kaplan-Meier estimations with log rank testing, and Cox proportional hazard model analyses were used to characterize the influence of treatment plan margins on overall survival. Where appropriate, post hoc Bonferroni-Holm corrections were employed.

## Results and discussion

### Commissioning of the SARRP unit and measurement of depth dose curves

Depth dose measurements were performed for different beam collimation settings (X-ray tube settings 220 kV, 13 mA, 0.15 mm copper filter) with a small animal phantom consisting of stacked 1 mm polystyrene plates and are shown exemplarily for 5× 5 mm^2^ collimation (Fig. 1a and b). Thermoluminescence dosimeters with rod-like or microcube-like shape or GAFchromic EBT3 films were used, respectively, in discrete and continuous measurement set-ups (Fig. 1b-d). The obtained data were compared to the commissioning data provided by the supplier as measured or as calculated with the point dose calculator tool PDC 1.2 (X-Strahl) modelling the dimensions and the material of our inhouse phantom, respectively. The different dosimetry techniques provided highly comparable results with TLD rods revealing the strongest variance – most likely attributable to suboptimal positioning in the diagonal of the irradiated field, since the phantom was aligned by vision control according to the laser coordinate system of the SARRP. Good agreement with the supplier’s commissioning data as measured was observed for up to 7 mm in depth. However, beyond 10 mm depth the observed deviation exceeded 4% (Fig. 1c-f). Discrepancies in backscatter conditions originating from phantom size and material apparently were responsible for these differences [16], because they were largely abrogated when the characteristics of our inhouse phantom were modelled into the supplier’s depth dose curve (Fig. 1e). The lateral penumbra measurements indicated adequate beam collimation which was comparable to previously reported results [14] and performed in x-axis slightly better than in y-axis (Fig. 1f).

### Contrast-enhanced and native CBCT scans for tumor localization, tumor volume follow-up, treatment planning, dose administration, and repositioning of animals

Seven days after intracranial inoculation of GL261 glioblastoma cells, tumor volume follow-up by contrast-enhanced CBCT scans with the SARRP unit was started (Fig. 2a and b). All tumors were well detectable, and length by height by width (Lx Hx W) calculation revealed contrast enriching volumes of 7 mm^3^ in average (Fig. 2b and c). Animals were randomized to three radiotherapy treatment groups with different beam collimation settings (5× 5, 3× 3, or 3× 9 mm^2^ collimation, respectively) and an untreated control group. No significant differences in tumor volumes were observed between animals randomized to different treatment arms at the start of therapy on d7 (Fig. 2c). Of note but not surprisingly, the strongest variation in tumor aspects was observed along the inoculation axis ranging from < 1 to > 5 mm (Fig. 2d). For treatment planning, the isocenter was assigned to the center of the contrast enriching volume, and two transversal, contralateral beams (gantry positions − 90° and 90°) of 5× 5, 3× 3, or 3× 9 mm^2^ collimation (fixed nozzle tube collimators), respectively, were defined. Dose volume histograms of one representative animal show that the tumor volume was well covered with the 5× 5 and 3× 9 mm^2^ collimation settings (Fig. 2e). In case of 3× 3 mm^2^, however, the tumor aspect along the inoculation axis (H-axis) exceeded 3 mm already at d7, and thus the tumor volume was not fully covered with dose.

**Fig. 4:**
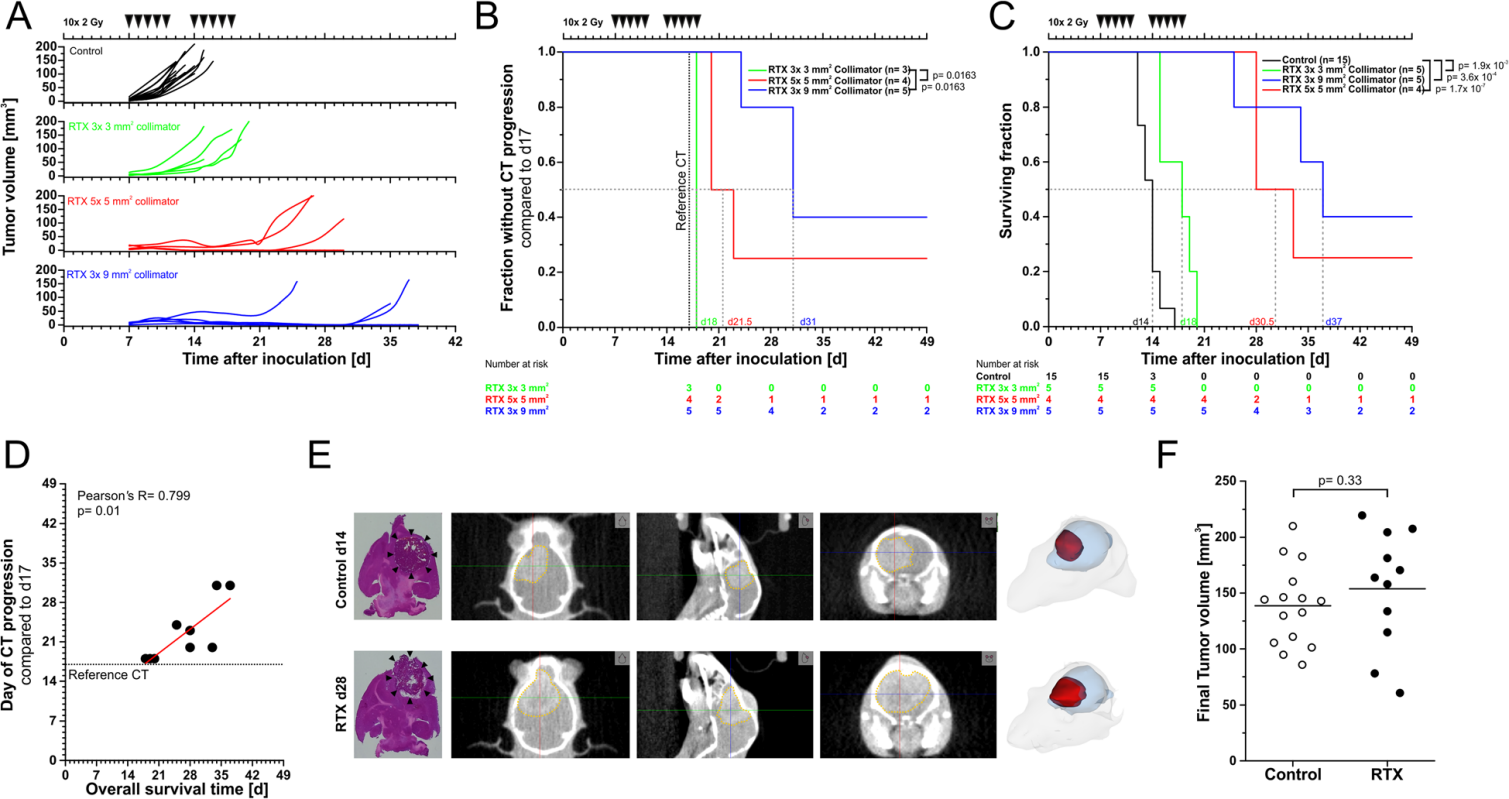
Therapeutic efficacy, local control, and overall survival. **a** GBM tumor growth in control animals and animals irradiated according to the treatment plans shown in Fig. 2e as determined by Lx Hx W volumetric method depicted in Fig. 3a. **b** Time-to-CT-progression analysis derived from tumor volumes as depicted in (**a**). Tumor volumes determined on d17 served as reference. *p*-values were derived from log rank tests and were subjected to Bonferroni-Holm multiple testing correction. **c** Kaplan-Meier survival analysis of animals in the described treatment groups. *p*-values were derived from log rank tests and were subjected to Bonferroni-Holm correction. **d** Pearson correlation analysis of CT progression and overall survival as shown in (**b**) and (**c**). **e** Exemplary contrast-enhanced CBCT scans of a control mouse (upper panel) and a mouse treated with 10 × 2 Gy (5× 5 mm^2^ collimation, lower panel) when moribund. 40 μm H&E stained tissue sections (left) of brains in intersecting planes similar to the ones shown in the CBCT scans. Arrowheads indicate the tumor. 3D volume renderings (right) display similar tumor size (red), shape, and localization within the brain (blue). **f** Tumor volumes of moribund control and radiotherapy-treated animals. p-value as calculated by exact Wilcoxon Rank test

The three treatment plans were administered to three randomized groups of animals in order to analyze the impact on tumor growth and overall survival. Contrast-enhanced CBCT scans were performed on d7, d10, d14, and d17. On all other days, isocenter alignment was inferred from the relative position to bony structures in native CBCT scans (Fig. 2b). Ten daily fractions of 2 Gy were administered over 2 weeks (2 × 5 × 2 Gy, Fig. 4a).

In-depth analyses of the margins between the contrast enriching tumor volumes and the respective collimation settings of all irradiated animals over time showed that in the 5× 5 and 3× 9 mm^2^ collimation treatment groups margins of ≥0.7 mm in L-axis and ≥ 1.3 mm in H-axis were achieved, except for only one animal in the 3× 9 mm^2^ group (Fig. 2f). In contrast, in the 3× 3 mm^2^ collimation treatment group, tumor L-axis aspects of all animals exceeded the collimator extensions (3 mm) in the second week of treatment, and for all but 2 animals tumor H-axis aspects were larger than 3 mm already in the first week of treatment. Accordingly, no proper margins were accomplished in this treatment group. With respect to sparing the normal brain tissue, 3× 9 mm^2^ collimation clearly achieved better results than 5× 5 mm^2^, since only approximately 20% of normal brain received ≥1.8 Gy per fraction vs. approximately 30% in case of 5× 5 mm^2^ (Fig. 2e). It should be noted that multi-beam approaches with more than 2 beams and particularly continuous arc radiation protocols which are supported by the SARRP device should further reduce the dose that is delivered to the normal brain. Yet, since convincing tumor volume coverage was obtained and in view of feasibility and throughput, we decided to use the rather simple two-beam strategy.

For follow-up monitoring, tumor volumes were determined in contrast-enhanced CBCT scans by two different approaches: By Lx Hx W measurement of the 3 longest orthogonal axes assuming a box-like shape, and – more accurately – by manual contouring with the segmentation editor tool of ImageJ software [15] (Fig. 3a). A clear and significant correlation was observed for the volume data obtained via these two approaches for both, irradiated and non-irradiated tumors (Fig. 3b). Nevertheless, as to be expected Lx Hx W measurements resulted in significantly larger tumor volumes, because the longest aspects were measured (Fig. 3c). Importantly, for the majority of tumors, the differences in the calculated volumes were very small (median < 5 mm^3^), and there was no significant difference between irradiated and non-irradiated tumors (Fig. 3d) indicating that the imprecision of assuming box-like shapes with the Lx Hx W method was similar for untreated and irradiated tumors. Given that for very large cohort analyses with relevant numbers of serial CBCT scans, manual contouring would require a substantial amount of time (approximately 12 min per tumor volume vs. 1 min for Lx Hx W calculation), this error appears of rather minor importance. Yet, automated contouring tools would be very helpful, particularly for GBM models with more invasive growth patterns. Overall, contrast-enhanced CBCT scanning represents a versatile technique for tumor localization and volume follow-up. At the same time it helps to reduce animal numbers in the sense of the 3Rs of animal welfare, since serial scans of individual animals can be performed [17].

### Treatment efficacy and feasibility

Radiation with all three collimation settings exerted well detectable effects on tumor growth (Fig. 4a). However, whereas 3× 3 mm^2^ collimation resulted in rather marginal growth delay, 5× 5 mm^2^ and 3× 9 mm^2^ collimation did achieve objective local control, yet to different extents. The median timepoint of progression in CBCT was d31 for 3× 9 mm^2^ collimation vs. d21.5 for 5× 5 mm^2^ collimation. In case of 3× 3 mm^2^ collimation, clear local control was not observed, and 2/5 animals needed to be sacrificed due to neurological symptoms even before the end of the radiation treatment (Fig. 4b). These observations basically translated into overall survival, with 3× 9 mm^2^ collimation revealing the best results, including 2/5 animals which were tumor-free at the end of the experiment (d50). Accordingly, Pearson correlation analysis revealed a statistically significant positive correlation between the timepoint of CBCT progression and overall survival time suggesting that CBCT progression may be used as a surrogate endpoint in order to spare animals from neurological symptoms and unnecessary suffering. Of course, this would only be applicable in case of therapeutic approaches in which local control is the foremost goal and/or the major determinant of overall survival as is the case for GBM.

Not surprisingly, the treatment success was clearly dependent on the size of the margins between the contrast enriching tumor volumes and the respective beam collimation settings. So, the two animals in the 3× 3 mm^2^ collimation group with the best therapeutic outcome were the ones with the largest treatment margins (Fig. 2f). Conversely, the single animal in the 3× 9 mm^2^ collimation group with particularly rapid progression and poor overall survival was the one whose tumor L-axis aspect exceeded 3 mm already in the first week of treatment (Fig. 2f). Detailed Cox proporational hazard model analyses revealed that for the chosen treatment regimens particularly the H-axis margins affect overall survival, both as means over treatment time (d7, d10, d14, d17) and as single values at the first day of radiotherapy (d7) (Table 1).

**Table 1:** Univariate Cox proportional hazard model of overall survival and margins between contrast enriching tumor volumes and beam collimation settings

Variable (Univariate analyses)	Hazard ratio (95% Confidence interval)	*P*-value	*P*-value adjusted (Bonferroni-Holm)
Margin H-axis mean over time (d7, d10, d14, d17) [mm]	0.53 (0.35–0.81)	0.0034	0.0136
Margin H-axis d7 [mm]	0.67 (0.50–0.90)	0.0088	0.0264
Margin L-axis mean over time (d7, d10, d14, d17) [mm]	0.50 (0.26–0.93)	0.0284	0.0568
Margin L-axis d7 [mm]	0.65 (0.32–1.32)	0.2318	0.2318

Margin sizes between contrast enriching tumor volumes and beam collimation settings in H- and L-axis [mm] as means over time (d7, d10, d14, d17) or as single values of d7 and overall survival times [d] were subjected to univariate Cox proportional hazard model analyses and post-hoc Bonferroni-Holm correction

Exemplary contrast-enhanced CBCT scans including volume renderings and matching 40 μm H&E histological sections are shown in Fig. 4e. When reaching the pre-defined humane endpoints, tumors of irradiated animals as compared to non-irradiated controls displayed slightly distorted aspect ratios with cranial-caudal extension and less clearly defined boundaries to the neighboring normal tissue, suggesting tumor cell migration out of the irradiated field as has been described by others [18, 19]. However, this requires more in-depth investigation. Notably, tumor volumes of moribund animals were not significantly different between irradiated and non-irradiated groups (Fig. 4f).

### Toxicity of contrast medium administration and irradiation

**Fig. 5:**
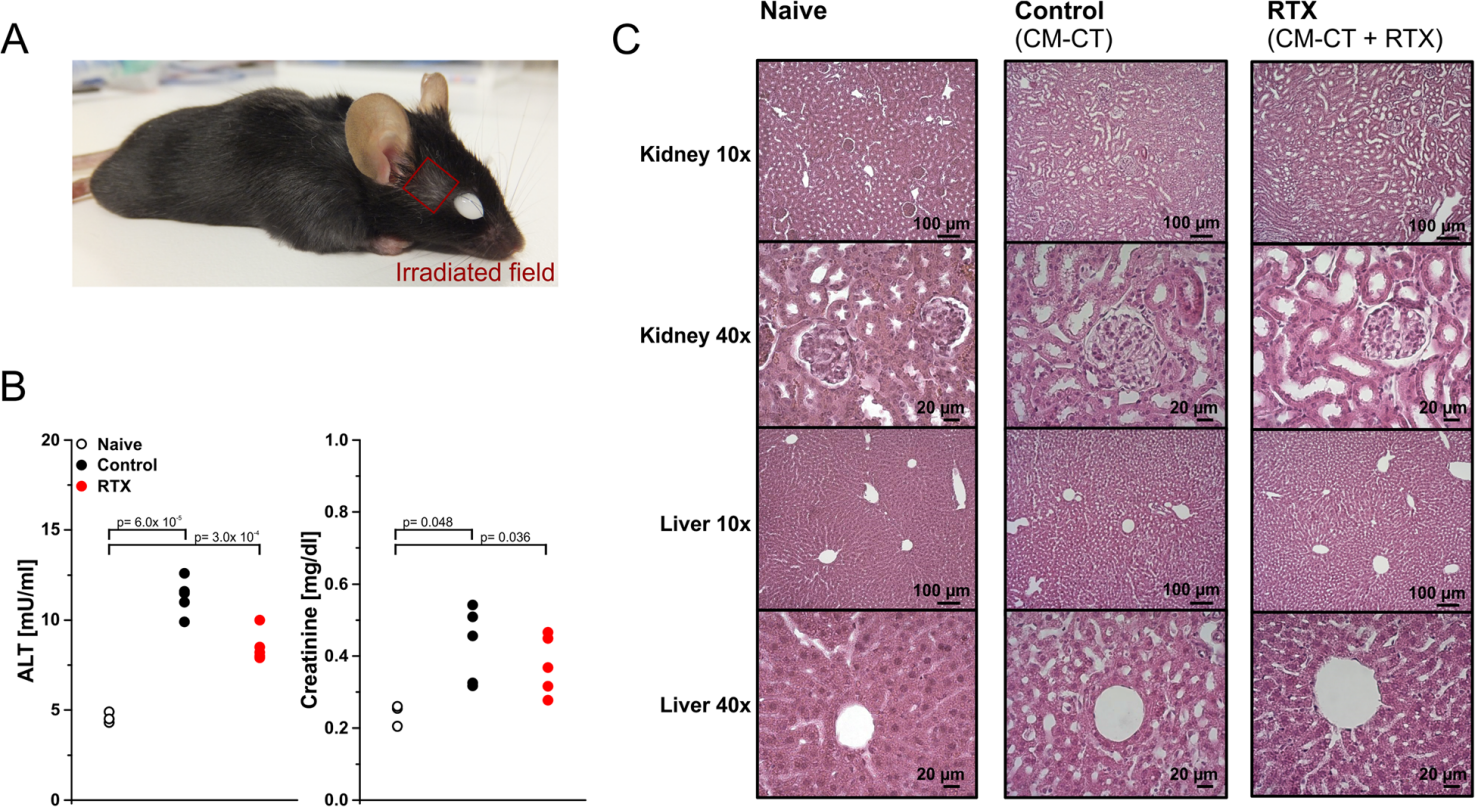
Tolerability of contrast medium application and irradiation. **a** Premature spot baldness in the irradiation field. **b** Serum alanine aminotransferase activities in naïve animals, control animals (only contrast-enhanced CT scans), and radiotherapy-treated animals (contrast-enhanced CT scans plus irradiation) after 2 weeks of therapy (d18). p-values were calculated by Student’s t-test with Bonferroni-Holm correction. **c** Corresponding histological analyses of exemplary 10 μm sections of livers and kidneys (H&E staining, light microscopy at 10x and 40x magnification). Scale bars as indicated

The treatment procedure, including repeated anaesthesia, contrast agent administration, and irradiation, was well tolerated by all animals. Nevertheless, premature spot baldness and depigmentation deriving from damaged hair follicles were observed in the irradiated areas as expected (Fig. 5a). In order to assess potential systemic toxicities of repeated contrast medium administration, serum creatinine and alanine aminotransferase (ALT) activity levels were assessed after the second week of therapy (d18). As compared to completely naïve controls, animals in our experimental groups displayed significantly enhanced but subclinical ALT and creatinine serum levels. No other indicators of relevant liver and/or kidney toxicity, including histopathological alterations or overall weight loss, were observed (Fig. 5c), suggesting that the increase in ALT and creatinine serum levels originated from acute toxicity reactions, most likely due to the hemodynamically relevant volume of intravenous contrast medium injection that was needed in order to achieve good radiological contrast (300 μl).

Here, we provide proof-of-concept for contrast-enhanced, CBCT-based, fractionated radiotherapy and follow-up monitoring of orthotopic mouse glioblastoma with the help of a stand-alone small animal radiotherapy platform with integrated CBCT scanner. The radiation regimen closely resembling glioblastoma radiotherapy in the clinical routine is feasible, well tolerated, and may serve as a basis for combined modality treatment approaches with biologically targeted and/or immunotherapeutic agents in the context of preclinical target validation [20, 21]. No other imaging modalities, such as MR, PET, or BL imaging, are needed for tumor localization, treatment planning, dose administration, and/or tumor volume follow-up. But if available, they can be integrated [5, 6, 9, 22].

Our study relies on intracranial implants of syngeneic GL261 cells. We have also tested orthotopic xenotransplants of established human glioblastoma cell lines in immunocompromised mice with similar experiences (data not shown). However, the application of this methodology for orthotopic transplants of patient-derived, low-passage-number, glioma stem-like cell isolates which commonly show more invasive growth patterns will require further investigation [20, 21, 23, 24].

## Conclusions

Contrast-enhanced, CBCT-based, fractionated radiation of orthotopic mouse GBM represents a versatile tool for the development and evaluation of multimodal radiotherapeutic approaches with novel compounds. In principle, this method is not only applicable for GBM but also for other orthotopic cancer models and may therefore be instrumental to accelerate the transfer of in vitro acquired results into clinical testing.

## Data Availability

The datasets generated and analyzed during the current study available from the corresponding author on reasonable request.

## Abbreviations

ALT: Alanine aminotransferase
BLI: Bioluminescence imaging
CBCT: Conebeam computed tomography
CT: Computed tomography
FCS: Fetal calf serum
GBM: Glioblastoma
MRI: Magnetic resonance imaging
PBS: Phosphate-buffered saline
PET: Positron emission tomography
TLD: Thermoluminescence dosimeter
